# Chromosomes missegregated into micronuclei contribute to chromosomal instability by missegregating at the next division

**DOI:** 10.18632/oncotarget.26853

**Published:** 2019-04-12

**Authors:** Bin He, Nisha Gnawali, Albert W. Hinman, Aaron J. Mattingly, Alyssa Osimani, Daniela Cimini

**Affiliations:** ^1^ Department of Biological Sciences, Virginia Tech, Blacksburg, VA 24061, USA; ^2^ Biocomplexity Institute, Virginia Tech, Blacksburg, VA 24061, USA; ^3^ Current affiliation: Orlando Health, MP 401, Orlando, FL 32819, USA; ^4^ Current affiliation: Department of Genetics, Stanford University School of Medicine, Stanford, CA 94305, USA; ^5^ Current affiliation: Department of Cell and Tissue Biology, UCSF, San Francisco, CA 94122, USA

**Keywords:** micronucleus, Chromosome, missegregation, lagging chromosome, mitosis

## Abstract

Micronuclei (MNi) are extranuclear DNA-containing structures that form upon mitotic exit from unsegregated chromosome fragments or anaphase lagging (whole) chromosomes (LCs). MNi formed from whole chromosomes are of particular interest because LCs are observed in both cancer and non-cancer cells, and are recognized as a major source of chromosomal instability (CIN) in cancer cells. Here, we generated a PtK1 cell line expressing a photoactivatable H2B histone to study the behavior of whole chromosome-containing MNi at the mitosis following their formation. Importantly, MNi of PtK1 cells did not display the membrane rupture or transport defects reported for other cell types. Despite this, we found that most micronucleated cells displayed some kind of chromosome segregation defect and that the missegregating chromosome was the one derived from the MN. Moreover, condensation of the chromosome within the MN was frequently delayed and associated with failure to align at the metaphase plate. Finally, the defective condensation of the MN-derived chromosomes could also explain the frequent occurrence of cytokinesis failure in micronucleated cells. In summary, we find that chromosomes from MNi may trigger a CIN phenotype by missegregating at the mitosis following MN formation.

## INTRODUCTION

Segregation of sister chromatids to opposite spindle poles during mitosis ensures equal chromosome distribution between the daughter cells. This is achieved through the attachment of sister chromatids to microtubules from opposite spindle poles in early mitosis. This attachment occurs at a specialized proteinaceous structure known as the kinetochore (KT), which assembles at the centromeric region of the chromosome. Individual sister chromatids can establish incorrect merotelic KT attachment by binding microtubules from both spindle poles [1]. When persisting through anaphase, some of these merotelically attached chromosomes lag behind at the spindle equator while all the other chromosomes move to the spindle poles [2, 3], and for this reason they are typically referred to as anaphase lagging chromosomes (LCs). Upon mitotic exit, a LC forms a micronucleus (MN) enclosed in its own nuclear envelope separated from the main nucleus [4]. Chromosome fragments present at mitosis are also unable to move to the spindle pole and form micronuclei (MNi) upon mitotic exit. Simple microscopy-based techniques can be used to identify MNi [5] and combination of the MN assay with immunostaining for KT proteins can be used to easily discriminate DNA fragment- and LC-derived MNi [6]. Because of the ease of detection, MNi are widely used as indicator of genetic damage, and high frequencies of MNi are found in cells with DNA repair or cell cycle checkpoint defects and in cells exposed to radiations or DNA damage-inducing chemicals or toxins [5, 7]. Moreover, the frequency of MNi increases with age and is a biomarker for increased cancer risk [8, 9].

Despite the ample use of MN analysis as a measure of genetic damage, the basic biology of MNi and their impact on cell function are not fully understood. Some studies have reported higher rates of cell death in micronucleated (MNed) vs. non-MNed cells [10, 11]. However, the MNi in these studies were induced by ionizing radiation and replication stress, respectively, thus raising the possibility that the increase in cell death may be due to high levels of genome-wide DNA damage and not simply to the presence of MNi. Other studies have shown that a number of cellular functions, including DNA replication, transcription, and DNA repair, are defective within the MN [12–14]. These defects were suggested to be, at least in part, caused by defective nuclear import due to defective assembly of the nuclear envelope/nuclear pore complex around the MN [12, 13, 15]. Moreover, the nuclear envelope of MNi in certain cell types was shown to frequently collapse [16] or assemble improperly [15, 17], possibly explaining chromothripsis-like phenomena found concomitantly with MNi [12, 18]. Finally, a number of reports used live-cell imaging to show that MNi can persist through subsequent cell cycles [10–12, 19, 20].

The impact of whole chromosome-containing MNi on cell fate is of particular interest because such MNi originate from LCs, which represent the most common chromosome segregation defect found in chromosomally unstable cancer cells [21, 22]. Chromosome fragments, which would also give rise to MNi, are rarely observed in mitotic cancer cells [21]. Although LCs are viewed as the major cause of chromosomal instability (CIN) in cancer cells [21, 22], a majority of LCs were shown to end up in the correct daughter of mitotic human colorectal cancer cells [23], raising the question as to whether LCs have a real impact on CIN. Here, we addressed this question by examining chromosome segregation at the mitosis following the formation of whole chromosome-containing MNi in live PtK1 cells. The choice of this experimental system was mainly dictated by the observation that newly formed whole-chromosome MNi in PtK1 cells display minimal-to-no defects in nuclear envelope integrity or transport of DNA damage response proteins. Thus, this experimental system offers the opportunity to study the fate of MNi independent of the defects associated with nuclear envelope functions typical of experimental systems used in previous studies.

## RESULTS

**Figure 1:**
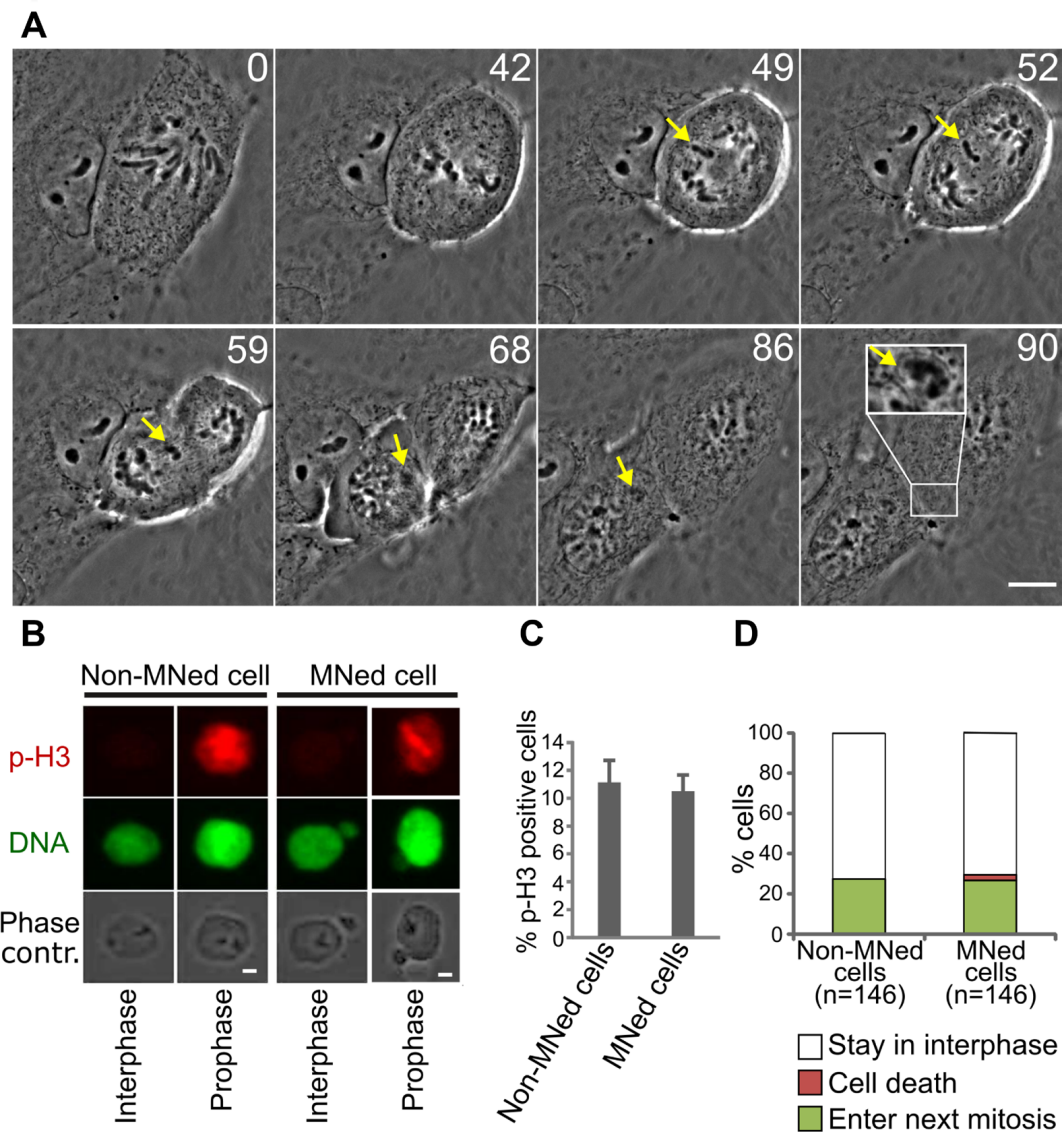
MNed PtK1 cells progress through the cell cycle (**A**) Still images from time-lapse phase contrast movie showing formation of whole chromosome-containing MN in PtK1 cells after STLC washout (see Supplementary Video 1). Yellow arrows indicate the LC and the MN it forms upon mitotic exit. Inset in the last frame shows a 2X enlargement of the newly formed MN. Time stamps represent elapsed time in minutes. Scale bar, 10 μm. (**B**) pH3 immunostaining (red) used to identify MNed prophase cells. MNi in prophase cells were identified by DAPI staining (green) and phase contrast microscopy. Scale bar, 2.5 μm. (**C**) Quantification of pH3-positive MNed and non-MNed cells showing similar numbers of prophase cells within the two subpopulations. The data reported represent the mean ± s.e.m. calculated from three independent experiments in which a total of 1319-1351 cells were analyzed, with 98-114 cells being MNed. (**D**) Quantification of proliferation and death rates in MNed and non-MNed cells obtained by long term time-lapse phase contrast microscopy (see Supplementary Video 2).

To induce MNi containing whole chromosomes, we used an STLC-washout protocol (see Materials and Methods for details), which increases the frequency of LCs, which in turn form MNi upon mitotic exit [4] (Figure 1A; Supplementary Video 1). To avoid prolonged mitotic arrest, which is known to induce DNA damage [24], the STLC treatment was limited to 3 hours. This experimental design allowed us to specifically study the fate and mitotic behavior of micronucleated (MNed) PtK1 cells at the mitosis following formation of LC-derived MNi.

### Cells with whole-chromosome MNi are as likely as non-MNed cells to enter mitosis

First, we asked whether MNed cells re-entered mitosis after MN formation. To this end, we fixed cells 24 hours after STLC washout and determined the fraction of MNed vs. non-MNed cells re-entering mitosis by identifying cells positive for phosphorylated histone H3 (p-H3) staining (Figure 1B), but in which the nuclear envelope had not yet broken down. We found that this “prophase index” was similar between MNed and non-MNed cells (Figure 1C), thus indicating that the fraction of MNed cells re-entering mitosis was sufficiently large to allow analysis of MNed cells at the mitosis following MN formation. Consistent with this, long-term live cell imaging showed that the percentage of MNed cells re-entering mitosis during imaging was similar to that of randomly sampled non-MNed cells (27.4% and 26.7%, respectively; Figure 1D and Supplementary Video 2). Moreover, we found that only a very small portion of MNed cells died (2% in interphase and 0.7% in mitosis) during imaging (Figure 1D). Although these rates of cell death are higher than those observed in non-MNed cells (in which cell death was never recorded during imaging), they are much lower than those observed in cells with DNA damage-induced MNi [10, 11], suggesting that the cell death observed in these previous studies was likely due to widespread DNA damage and not to the presence of a MN.

**Figure 2:**
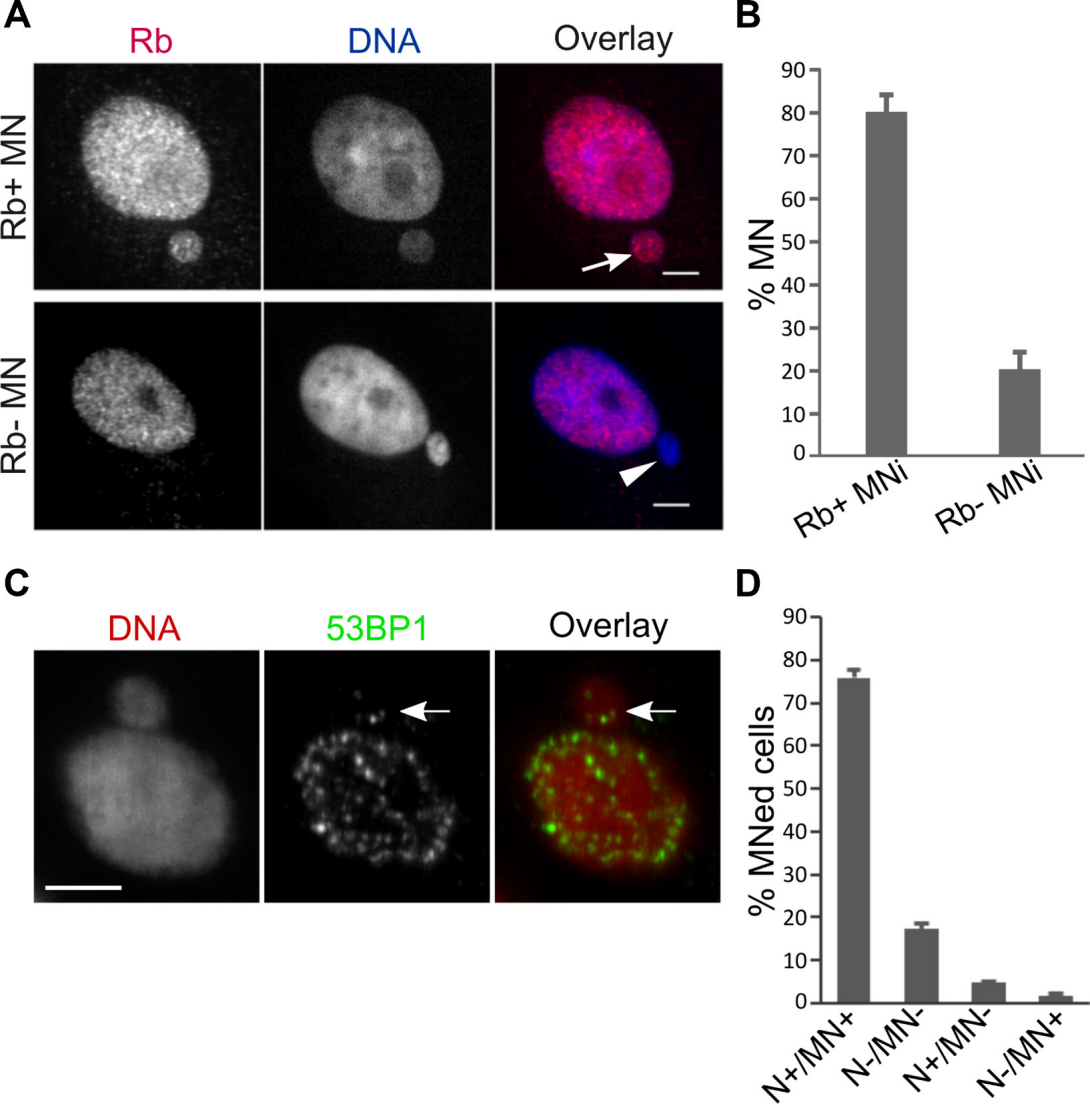
PtK1 cell MNi do not experience rupturing of the nuclear membrane and can recruit DNA damage signaling proteins (**A**) Loss of nuclear localization of the retinoblastoma (Rb) protein is used as a marker for nuclear envelope rupture [16]. Intact (arrow) and ruptured (arrowhead) MN in fixed PtK1 cells immunostained for Rb (left/red). The RB- MN is clearly visible by DAPI staining (middle/blue). Scale bar, 5 μm. (**B**) Quantification of Rb+ vs. Rb- MNi shows that most of the MNi maintained nuclear localization of Rb protein, which is indicative of intact nuclear envelope. The data represent the mean ± s.e.m. calculated from three independent experiments in which 179-291 MNed cells were analyzed. (**C**) Example of MNed cell with 53BP1 localization both in the nucleus and within the MN. DNA damage was experimentally induced by exposing cells to Bleocin for 3 hours prior to fixation. Scale bar, 5 μm. (**D**) Quantification of 53BP1 immunostaining data. MNed cells were grouped in different categories depending on whether both the main nucleus and the MN (N+/MN+), neither the main nucleus nor the MN (N−/MN−), the main nucleus but not the MN (N+/MN−), or the MN but not the main nucleus (N−/MN+) displayed 53BP1 foci. The data represent the mean ± s.e.m. calculated from two independent experiments in which 324 and 348 MNed cells were analyzed, respectively.

Next, we examined the integrity of the nuclear envelope in our experimentally-induced whole chromosome MNi, to exclude the possibility that the membrane of such MNi was collapsing/rupturing, as previously reported for human cell MNi [16]. As a measure of nuclear membrane integrity, we assessed the presence of the retinoblastoma protein (Rb) (Figure 2A), which was previously shown to be lost from MNi undergoing nuclear membrane rupture [16]. We found that most MNi retained Rb staining (Figure 2B), indicating that most of our whole-chromosome MNi did not experience membrane collapse/rupture. Finally, we determined that, as opposed to what was found for human cells [12, 15], the vast majority of MNed PtK1 cells were able to recruit the DNA damage response marker 53BP1 inside the MN in response to experimentally-induced DNA damage (Figure 2C–2D). Overall, these data indicate that after formation of whole chromosome-containing MNi, MNed PtK1 cells can re-enter mitosis with intact MNi that do not display the severe membrane defects observed in other cell types [12, 16]. As such, this PtK1 cell-based experimental system can be used to study the behavior of MNed cells at the mitosis following MN formation without the confounding effects of DNA damage resulting from disruption of the MN membrane.

### MNed cells display high rates of chromosome segregation errors and further MN formation

**Figure 3:**
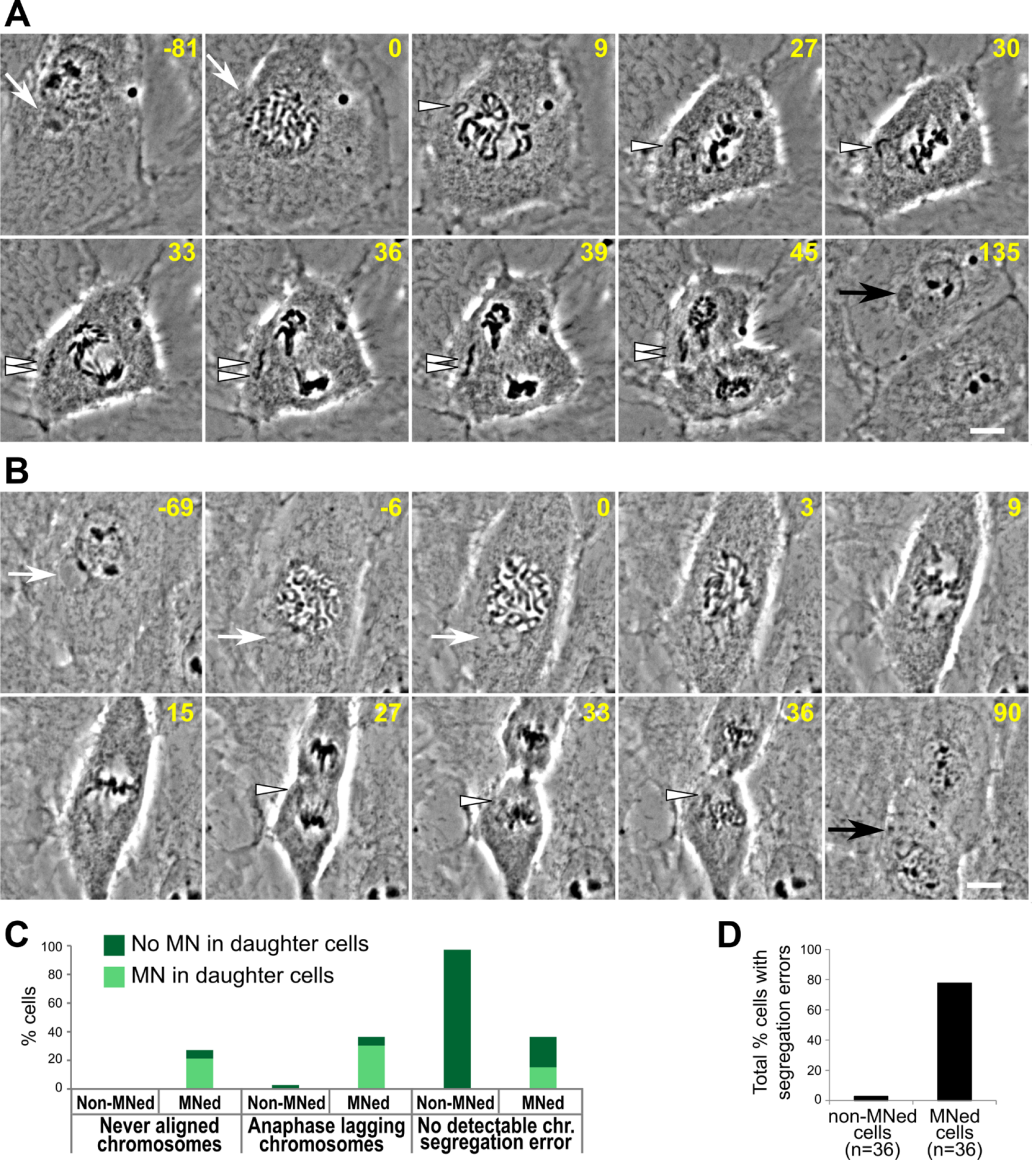
High rates of chromosome segregation errors in MNed cells (**A**–**B**) Representative time-lapse phase contrast micrographs of MNed cells that enter mitosis and display chromosome segregation errors. White arrows point at the MNi; white arrowheads indicate missegregating chromosomes/chromatids; black arrows indicate the newly formed MN in one of the daughter cells. Scale bars, 10 μm. (A) MNed cell with a missegregating chromosome that never aligns at the metaphase plate. After anaphase onset, the sister chromatids appear to separate from each other, but fail to move to the spindle poles. Instead, they lag near the cell equator and end up in the same MN in one daughter cell (see also Supplementary Video 3). (B) In this cell, when the nuclear envelope of the main nucleus breaks down, the MN-derived chromosome (mnChr) appears under-condensed (see also Supplementary Video 4). The mnChr becomes mingled with the rest of the chromosomes, but at the end of mitosis a LC becomes visible and gives rise to a new MN in one of the daughter cells upon mitotic exit. (**C**) Quantification of chromosome segregation errors in live cell experiments. (**D**) Percentage of cells displaying at least one chromosome segregation error.

To study the behavior of MNed cells at the mitosis following MN formation, we used the data from our long-term time-lapse phase-contrast imaging experiments (Figure 1D) and examined the MNed and non-MNed cells that underwent mitosis during imaging (Figure 3A–3B; Supplementary Videos 3 and 4). We found that MNed cells frequently displayed chromosome segregation errors, including chromosomes that never aligned at the metaphase plate (Figure 3A, 3C) and chromosomes that aligned, but lagged behind at the spindle equator in anaphase (Figure 3B–3C). As a result of these chromosome segregation errors, in many cases (41.5%) MNi formed in one or both of the daughter cells (Figure 3A–3C). Moreover, MNi also formed in daughter cells of MNed cells undergoing mitosis with no detectable chromosome segregation errors (Figure 3C). Only a small portion (4 out of 36; ∼11%) of MNed cells segregated their chromosomes without visible defects and yielded daughter cells without MNi (Figure 3C). Overall, most MNed cells displayed chromosome segregation errors (Figure 3D) at the mitosis following MN formation.

### The MN-derived chromosome is responsible for the segregation errors

**Figure 4:**
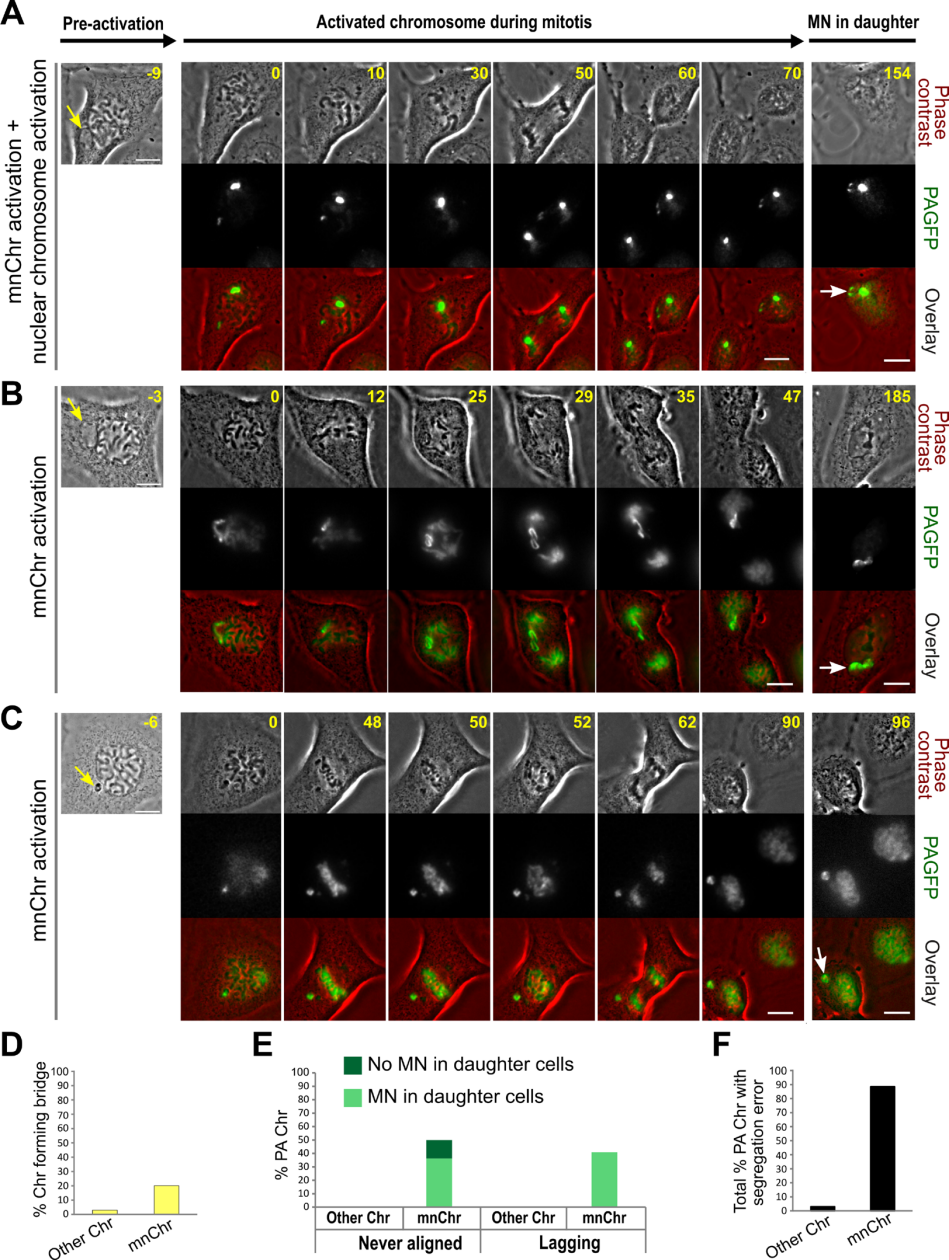
MN photoactivation shows that the mnChr is responsible for the segregation errors observed in MNed cells (**A**–**C**) Chromosomes in the main nucleus and/or in MNi (yellow arrow) were marked by photoactivation of H2B-PAGFP in PtK1 cells. Right panels in A-C show the MNed cell prior to photoactivation, and the yellow arrow points at the MN. The remaining panels show still images from time-lapse movies acquired after fluorescence photoactivation, with the frame on the right corresponding to a late time point to show MN formation (white arrow) in one of the daughter cells. For each cell, phase contrast images are shown in the top row, fluorescence images are shown in the middle row, and the overlay is shown in the bottom row. Time stamps indicate elapsed time in minutes. Scale bars, 10 μm. (A) One small region (corresponding to one chromosome) within the main nucleus and the mnChr were activated in this cell. The images show that the chromosome from the main nucleus segregates correctly, whereas the sister chromatids from the mnChr lag behind, ending up in the same daughter cell and in the same MN (see also Supplementary Video 6). (B) The mnChr aligns at the metaphase plate and its sister chromatids separate at anaphase onset. However, they lag behind, co-segregate to the same daughter cell, and form two MNi upon mitotic exit (see also Supplementary Video 7). (C) The mnChr in this cell never aligns at the metaphase plate, persists in the same position throughout mitosis, and re-forms a MN in one of the daughter cells upon mitotic exit. (**D**–**E**) Quantification of various chromosome segregation errors in photoactivation experiments. “PA Chr” refers to photoactivated chromosomes; “Other Chr” refers to photoactivated chromosomes within the main nucleus of a MNed cell. (**F**) Percentage of photoactivated chromosomes displaying at least one segregation error.

**Figure 5:**
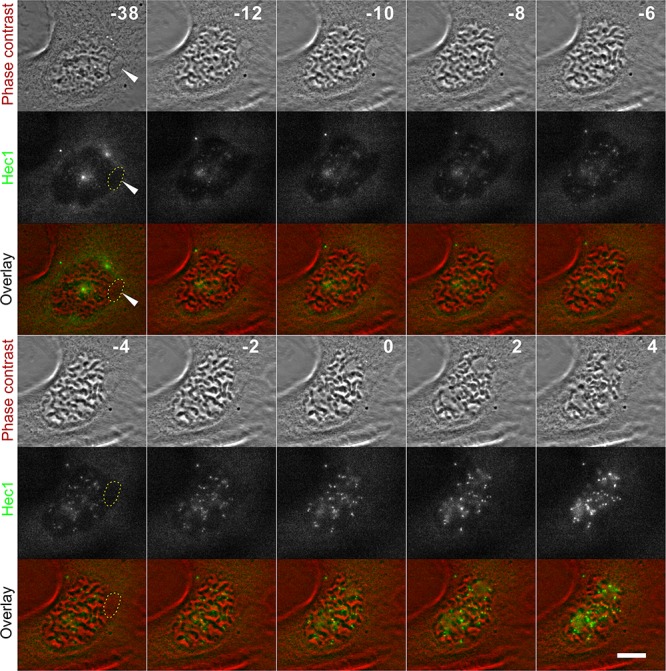
Hec1 loading in MN Hec1-GFP PtK1 cell with MN (white arrowhead) imaged during prophase. The white dotted line demarcates the MN boundaries in some of the frames. The time stamps indicate elapsed time in minutes, with 0 min corresponding to nuclear envelope breakdown. No Hec1 can be detected in the MN even at later time points (e.g., −2 min), when significant amounts of Hec1 are associated with the chromosomes within the main nucleus. The fluorescence images are maximum intensity projections of 5 focal sections imaged at 0.6 μm intervals. Scale bar, 5 μm.

Although in some cases it was possible to identify the MN-derived chromosome (mnChr) as the missegregating chromosome in MNed cells imaged by phase-contrast (Figure 3A), in many other cases, it was not clear whether the mnChr or some other chromosome was the one missegregating (Figure 3B). To address this, we generated a PtK1 cell line stably expressing photoactivatable-GFP-tagged H2B histone (H2B-PAGFP PtK1) and used it to specifically mark the entire MN (i.e., the mnChr). Preliminary experiments in which we activated the entire nucleus of prophase cells showed that photoactivation of H2B did not affect chromosome segregation (Supplementary Video 5). Moreover, as a control for MN photoactivation, we photoactivated a chromosomal region within prophase nuclei (Figure 4A; Supplementary Video 6) and found that missegregation of these nuclear chromosomes was very rare (Figure 4D–4F). To track the mnChr, we initially followed MNed cells by phase contrast and photoactivated the MN at the time of nuclear envelope breakdown (as detected for the main nucleus). In the vast majority of cases, the mnChr displayed some kind of segregation defect (Figure 4F; Supplementary Videos 6–7). These included anaphase bridges (Figure 4D), anaphase lagging (Figure 4E), and failure to align at the metaphase plate (Figure 4E). Some of these chromosome segregation defects were also associated with further MN formation upon mitotic exit (Figure 4E). It is important to note that in cells with MN photoactivation, missegregation of chromosomes from the main nucleus was extremely rare and limited to chromosome bridge formation (Figure 4D–4F), confirming that the chromosome missegregation events observed in MNed cells typically involve the mnChr and not chromosomes from the primary nucleus. In some cases, although the mnChr displayed apparently normal condensation (Figures 3A and 4B) and aligned at the metaphase plate (Figure 4B), upon separation, the two sister chromatids failed to segregate properly and ended up in the same daughter cell (Figures 3A and 4B), in some cases within the same MN (Figure 3A). This is a clear example of segregation of two sister chromatids to the same daughter cell, a phenomenon that is traditionally referred to as nondisjunction and leads to numerical chromosome aberrations in the daughter cells. In other cases, the missegregation event was even more obvious, as the mnChr did not align at the metaphase plate prior to anaphase onset. This behavior suggests that some mnChrs may be ineffective at mounting a mitotic checkpoint response, which typically delays anaphase onset until all chromosomes have become attached to microtubules [25]. Mitotic checkpoint proteins are diffusible [26] and therefore should be available to the mnChr as soon as the nuclear envelope breaks down. However, defective mitotic checkpoint signaling by the mnChr could be explained by defects in the assembly of the outer KT. Evidence of such defect was provided by the identification of live prophase MNed cells in which the outer KT protein Hec1 was not recruited to the MN at a time when it was clearly associated with the chromosomes within the main nucleus (example shown in Figure 5), consistent with recent findings by Soto *et al.*, who also found reduced levels of inner KT proteins and reduced levels of the mitotic checkpoint protein Mad1 on MN-derived chromosomes as compared to other chromosomes [19]. This observation also indicates that nuclear transport may be partly impaired, despite allowing for efficient import of certain proteins (e.g., 53BP1; Figure 2C–2D).

### Delayed condensation of the mnChr and cytokinesis failure in MNed cells

**Figure 6:**
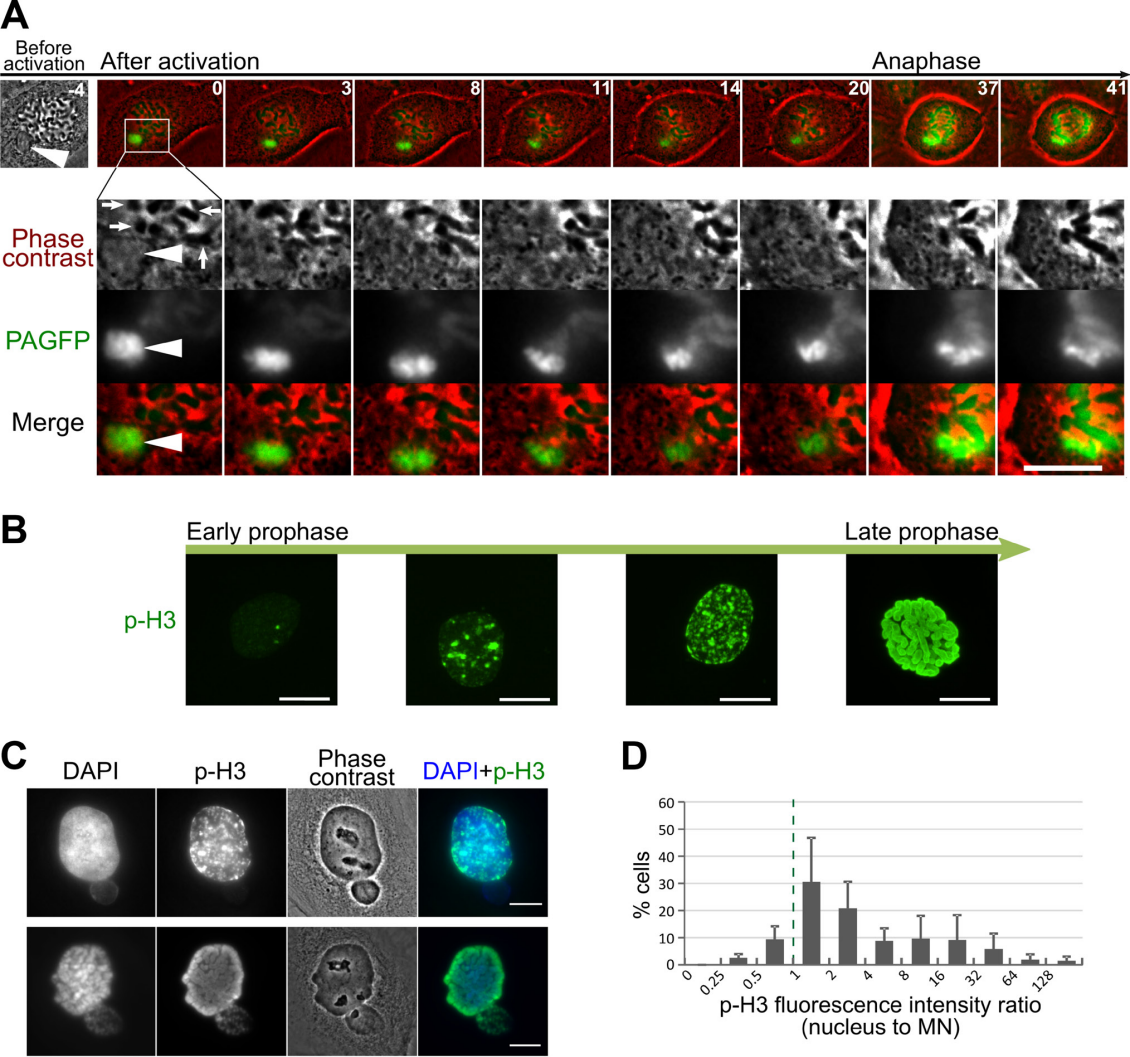
Delayed chromosome condensation of mnChrs (**A**) Photoactivated mnChr (white arrowhead) appears clearly under-condensed compared to the chromosomes in the main nucleus. The time stamps indicate elapsed time in minutes. At t = 0 min, the nuclear envelope of the main nucleus breaks down and the chromosomes (white arrows) are visibly condensed, as shown by their phase-dense appearance in the phase contrast images. However, at this time point, the mnChr does not appear condensed by phase contrast and the fluorescence of the activated PAGFP appears dispersed. During mitosis, the mnChr gradually condenses, but with a substantial delay that prevents its normal movement and segregation. Scale bars, 10 μm. (**B**) Fluorescence images of prophase cells immunostained for p-H3. The images show cells with progressively increasing levels of pH3 fluorescence. Cells with intense and widespread p-H3 staining also display significant chromosome condensation, indicative of late prophase stage. Scale bars, 10 μm. (**C**) p-H3 staining of MNed cells shows differences in fluorescence intensities between the MN and the main nucleus within the same cell. Scale bars, 10 μm. (**D**) Quantification of normalized (p-H3/DAPI) nucleus/MN fluorescence intensity ratio in MNed cells. The data, reported as mean ± s.e.m., are from three independent experiments, with a total of 218 MNed cells analyzed.

**Figure 7:**
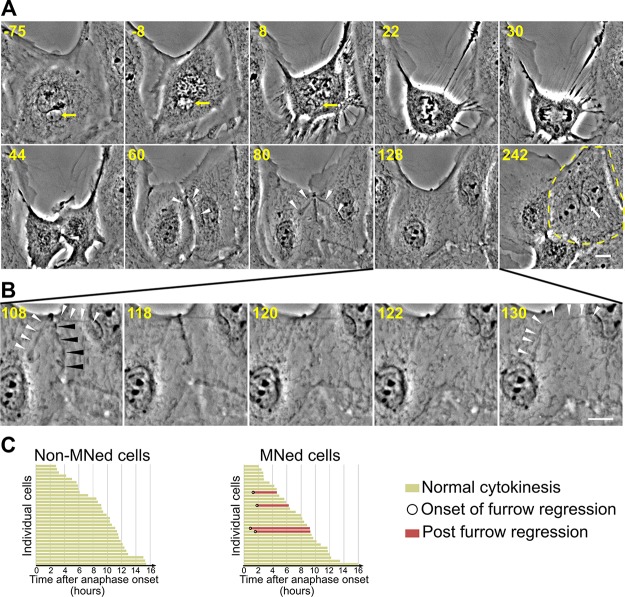
Furrow regression and tetraploidization in MNed cells (**A**) Phase contrast still images from time-lapse movie of a MNed PtK1 cell undergoing mitosis and in which an undercondensed chromosome becomes trapped by the cleavage furrow causing furrow regression, and hence cytokinesis failure and tetraploidy. Time stamps indicate elapsed time in minutes. Yellow arrows in the first three frames point at the MN and the mnChr. White arrowheads indicate the chromosome becoming trapped by the cleavage furrow starting at 44 min, and then becoming decondensed and stretched across the midbody region. Eventually, the cleavage furrow regresses, giving rise to a binucleate cell (marked by yellow dashed line in the 242 min frame) with MN (white arrow). Scale bar, 10 μm. (**B**) Close up view of the cleavage furrow region (black arrowheads) to highlight the time window during which furrow regression occurs. Note the quick disappearance of the cleavage furrow after the 118 min time point. At 130 min, the constriction between the two nuclei is substantially relaxed and the trapped chromatin (white arrowheads) has lost its association with the cell cortex and the midbody remnant. Scale bar, 10 μm. (**C**) Analysis of normal cytokinesis vs. furrow regression in MNed and non-MNed cells. Chromatin trapped in the cleavage furrow could, in some cases, be discerned in MNed cells exhibiting cleavage furrow regression. None of the cleavage furrows in non-MNed cells regressed.

In our live cell images, we noticed that the DNA in the MN frequently appeared less condensed compared to the chromosomes within the main nucleus prior to or at the time of nuclear envelope breakdown (see for instance −6, 0, and 3 min frames in Figure 3B and Figure 6A). In fact, in some instances, this undercondensation made imaging of mnChr in H2B-PAGFP PtK1 cells difficult, as the chromosome never became compact and bright enough to be easily visualized and imaging required high exposure times, which in turn resulted in the partial activation of the rest of the chromatin (Figure 4B–4C). In some cases, condensation of the mnChr increased after nuclear envelope breakdown, but did not reach the levels of condensation observed for chromosomes in the main nucleus (Figure 6A). These observations suggested that condensation of the mnChr was delayed compared to chromosomes in the main nucleus. To assess MN condensation, we quantified the fluorescence intensity of histone H3 Ser10 phosphorylation (p-H3) in the MN as compared to the main nucleus within individual MNed prophase cells immunostained for p-H3 (see methods section for details on quantification). As expected, the intensity of p-H3 gradually increased during prophase (Figure 6B), but in a majority of MNed cells, the levels of p-H3 were lower in the MN compared to the main nucleus (Figure 6C–6D). Specifically, ∼40% of prophase cells showed fluorescence intensity ratios (nucleus/MN) of ∼1, whereas in the remaining ∼60% of MNed cells, the level of p-H3 in the main nucleus was substantially higher than that found in the MN (Figure 6D). Thus, chromosome condensation is considerably delayed inside the MN compared to the main nucleus of MNed cells. Indeed, in some cases the mnChr persisted in an undercondensed state throughout mitosis (Figure 6A). This undercondensation could explain the increased rates of cytokinesis failure in MNed vs. non-MNed cells we observed in our phase-contrast live-cell imaging experiments (Figure 7A–7C). Indeed, in some MNed cells, the undercondensed mnChr persisted at the spindle equator as all other chromosomes moved to the poles (Figure 7A). The mnChr was unable to clear the spindle equator area upon furrow ingression, remained trapped by the cytokinetic furrow (Figure 7A–7B; Supplementary Video 8), and eventually resulted in furrow regression (Figure 7B). Interestingly, cytokinesis failure was specifically induced by lagging/unaligned mnChrs, but not by STLC-induced LCs. Indeed, none of the 59 “first-time” LCs we followed upon STLC washout (Figure 1A) became trapped at the cleavage furrow. Instead, these chromosomes, which displayed normal condensation, were displaced to one side of the ingressing furrow and formed MNi (Figure 1A), as previously described [4].

## DISCUSSION

**Figure 8:**
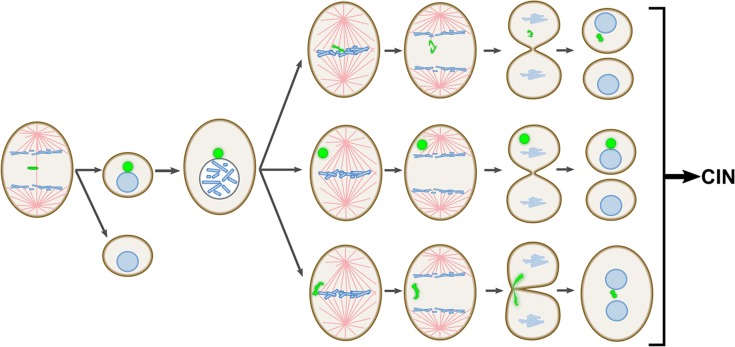
Schematic summary of the major findings of this study The diagram illustrates the propensity of mnChrs to missegregate at the mitosis following MN formation. Such propensity to missegregate makes MNi a major source of CIN.

In this study, we investigated the fate of newly formed whole chromosome-containing MNi in PtK1 cells, an experimental model that does not appear to suffer from rupture of the MN membrane or major defects in transport across the membrane. These phenotypes have been reported for several different human cell types [12, 13, 15–17] and can explain the abnormal mitotic behavior of chromosomes trapped in MNi in some of these cell types [12, 19, 20]. Despite the absence of these impairing defects of the MN membrane in PtK1 cells, the mitotic behavior of chromosomes derived from MNi (mnChrs) is highly abnormal. Indeed, our data show that mnChrs are particularly prone to missegregation and can display a variety of abnormal mitotic behaviors (see Figure 8 for summary of mnChr segregation errors observed in this study), including segregation of two sister chromatids to the same daughter cell (Figure 8), a phenomenon that is rarely observed when LCs arise for the first time [23], and trapping within the cleavage furrow (likely due to mnChr marked undercondensation), a behavior we never observed for “first-time” LCs. The trapping of mnChr in the cytokinetic furrow is particularly significant because it can lead to cytokinesis failure and tetraploidy, a condition known to contribute to CIN by increasing the rates of chromosome missegregation and/or the tolerance to aneuploidy [27–29]. Thus, by missegregating at the cell division following MN formation, whole chromosome MNi trigger a series of chromosome missegregation events that will lead to CIN (Figure 8), possibly explaining the previously reported correlation between MN burden and cancer risk in humans [8].

Although we found that transport across the MN membrane was sufficient for recruitment of 53BP1 in response to DNA damage, we also observed that recruitment of Hec1 to mnChrs was markedly delayed. Defective Hec1 recruitment could be due to suboptimal levels of inner KT proteins on the mnChr, an observation recently reported for MNi in RPE-1 cells [19]. However, this would more likely result in recruitment of low Hec1 levels, rather than a marked delay in recruitment. An alternative explanation would be that transport is simply delayed, as recently observed for MNi in RPE-1 cells [17]. A delay in key events associated with mitosis can also explain the delay in condensation of mnChrs in PtK1 cells. This finding adds to the observation that DNA replication can be still ongoing in the MN when the main nucleus is in the G2 phase of the cell cycle [12]. Because mitosis is completed over a much shorter time window than other cell cycle phases, delays in mitosis-specific events may have severe consequences, as there may not be sufficient time for such events to be completed before mitotic progression or mitotic exit. And if such events include a delay in assembly of the outer KT (Figure 5), then that KT would be unable to establish mitotic checkpoint signaling.

Our work focused on newly formed MNi specifically arising from merotelically attached LCs (most common chromosome segregation defect in cancer cells [21, 22]), as opposed to other studies that examined the behavior of MNi arising as a result of DNA damage [10, 11] or studies that analyzed MNi derived from other mitotic defects (e.g., unaligned chromosomes from CENP-E inhibition [19]) or a combination of mitotic defects (i.e., a mix of chromosome bridges, LCs, and unaligned chromosomes resulting from MPS1 inhibition [19]). Therefore, our experimental system is highly controlled, with MNi exclusively derived from LCs and no evidence for major disruption of the MN membrane. Despite this, the behavior of mnChrs at the cell division following MN formation is highly abnormal. Thus, it is clear that, regardless of their origin, the degree of membrane integrity, and the cell type in which they arise, MNi invariably lead to severe genome instability. Based on this, it is not surprising that MNi have been recognized as a biomarker of genotoxic damage and disease (particularly cancer) risk [30, 31].

## MATERIALS AND METHODS

### Generation of H2B-PAGFP PtK1 cell line

A PtK1 cell line stably expressing the H2B gene fused to PAGFP was produced through the transduction of retroviral particles according to the instructions for high-titer retrovirus production provided by Clontech. A H2B-PAGFPN1 plasmid was initially obtained as a kind gift from Dr. Jon Pines (The Gurdon Institute, University of Cambridge, UK). The H2B-PAGFP gene was then subcloned into the Not I and Hind III sites of the pLPCX retroviral vector (Clontech Laboratories, Inc.) that harbors the Ψ^+^ packaging sequence. In order to produce high efficiency, replication-incompetent, retroviral particles, packaging GP2-293 cells (Clontech Laboratories, Inc.), which carry the viral *gag* and *pol* genes, were transfected with the H2B-PAGFPpLPCX plasmid together with the pVSV-G vector (Clontech Laboratories, Inc.) that provided the viral envelope gene (*env*). The resulting retroviral particles were used to infect PtK1 cells that were subsequently placed under selection in Puromycin-containing media to obtain the final H2B-PAGFP cell line used in this study.

### Cell culture and treatment

PtK1, H2B-PAGFP PtK1, and HEC1-GFP PtK1 [32] cells were cultured in Ham’s F-12 media (Invitrogen) supplemented with 10% Fetal Bovine Serum (Invitrogen), 1 mM sodium pyruvate (Invitrogen), 14 mM sodium bicarbonate (Fisher Scientific), 1% antibiotic-antimycotic (Invitrogen), and maintained at 37° C in a humidified CO_2_ incubator. For live cell imaging, cells were either grown on glass-bottom dishes and imaged using a stage top incubator (Tokai Hit) or were grown on sterilized coverslips inside 35 mm Petri dishes, transferred into a modified Rose chamber [33] with top coverslip, and imaged on a microscope stage heated by an air stream incubator (Nevtek). To induce LCs and MNi, cells were incubated in 20 μM STLC (S-Trityl-L-cysteine; Sigma-Aldrich) for 3 hours. The drug was then washed out by rinsing the cells 4 times with warm media. Cells were then re-incubated in fresh Ham’s F-12 media for 24 hours before immunostaining or live-cell imaging. For live-cell imaging, cells were placed in phenol-free L-15 media (Gibco) with 4.5 g/liter glucose. To induce DNA damage, 21 hours after STLC washout, cells were treated with 50 μg/ml Bleocin™ (antibiotic from Streptomyces verticillus; Calbiochem) for 3 hours and then fixed and immunostained as described in the next section.

### Immunofluorescence staining, image acquisition, and image analysis

For histone H3 Ser10 phosphorylation (p-H3), Rb, and 53BP1 immunofluorescence, PtK1 cells were fixed in freshly prepared 4% formaldehyde for 20 minutes. Cells were then permeabilized in 0.5% Triton X-100 in PHEM buffer (60mM Pipes, 25mM HEPES, 10mMEGTA, 1mM MgSO4, pH 7.0) for 10 min. Cells were next incubated for one hour at room temperature in a blocking solution consisting of 10% boiled goat serum in PHEM buffer, followed by overnight incubation at 4° C in primary antibodies. Next, cells were subjected to four five-minute washes in PBS with 0.1% Tween 20 (PBST) and then incubated for 45 minutes at room temperature with secondary antibodies. Cells were finally washed (4 × 5 min) in PBST, stained with DAPI, washed again, and mounted in an antifade solution (90% glycerol, 10% Tris buffer, 0.5–1% n-Propyl gallate). The antibodies were all diluted in 5% boiled goat serum as follows: rabbit anti-phospho-(Ser10)histone H3 (Millipore), 1:100; mouse anti-Rb(4H1) (Cell Signaling Technology), 1:200; rabbit anti-53BP1 (Novus Biologicals), 1:500; Red-X-goat anti-rabbit (Jackson ImmunoResearch laboratories Inc.), 1:100; Red-X-goat anti-mouse (Jackson ImmunoResearch laboratories Inc.), 1:200; Alexa 488-goat anti-rabbit (Jackson ImmunoResearch laboratories Inc.), 1:300.

Imunostained PtK1 cells were imaged on a swept field confocal unit (Prairie Technologies) attached to a Nikon Eclipse TE-2000U inverted microscope. The microscope was equipped with a 100×/1.4 NA Plan-Apochromatic phase–contrast objective lens, phase–contrast transillumination, transmitted light shutter, and automated ProScan stage (Prior Scientific). The confocal head was accessorized with multiband pass filter set for illumination at 405, 488, 561, and 640 nm, and illumination was obtained through an Agilent monolithic laser combiner (MLC400) controlled by a four channel acousto-optic tunable filter. Digital images were acquired with a HQ2 CCD camera (Photometrics). Acquisition time, Z-axis position, laser line power, and confocal system were all controlled by NIS Elements AR software (Nikon Instruments Inc.) on a PC computer (Dell). Z-series optical sections through each cell analyzed were obtained at 0.6 μm steps.

**Figure 9:**
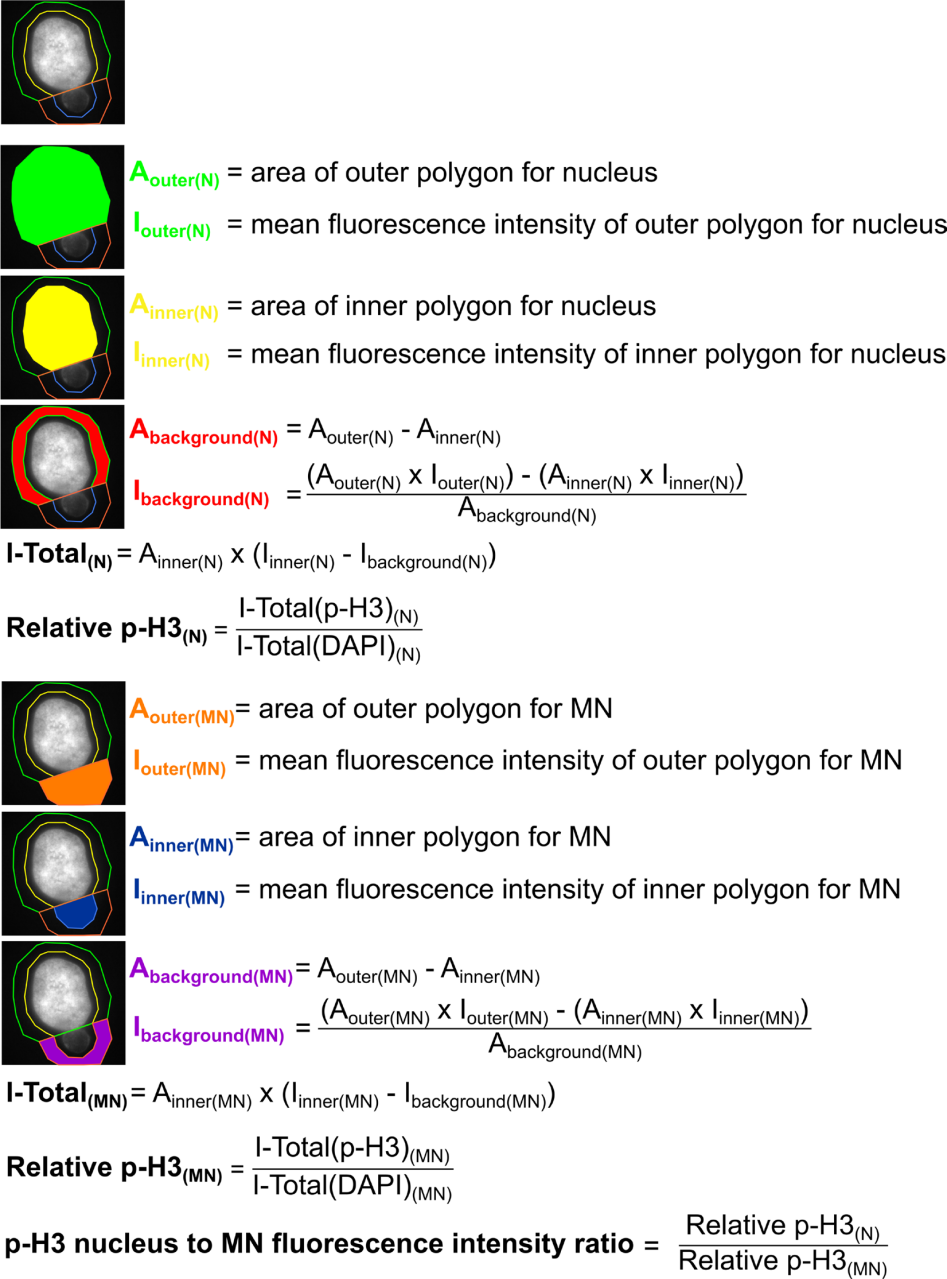
Description of the method used to quantify p-H3 fluorescence intensity for Figure 6D

Quantification of histone H3 phosphorylation (p-H3) was performed in ImageJ with background fluorescence intensity subtraction. For MN intensity, the DNA signal visualized by DAPI was used to draw a polygon (with area = A_Inner(MN)_ and mean fluorescence intensity = I_Inner(MN)_) approximately corresponding to the MN area. A larger outer polygon (with area = A_Outer(MN)_ and mean fluorescence intensity = I_Outer(MN)_) was traced around the MN polygon in an off-centered position, so that it would not overlap with the main nucleus and was traced in a way that it extended beyond the MN; the same thing was done for the main nucleus (see Figure 9, top diagram). Both DAPI and p-H3 fluorescence intensities were quantified for each MNed cell to obtain p-H3 fluorescence intensity relative to the DAPI fluorescence intensity. A nucleus to micronucleus ratio was then calculated and reported in Figure 6D. A detailed description of the method used for these fluorescence intensity quantifications is reported in Figure 9.

### Phase-contrast live-cell imaging

Phase-contrast live-cell imaging was performed on a Nikon Eclipse Ti inverted microscope equipped with phase–contrast transillumination, transmitted light shutter (Sutter Instruments), ProScan automated stage (Prior Scientific), and HQ2 CCD camera (Photometrics). Image acquisition, light shutter, and XYZ-axis positions were all controlled by NIS Elements AR software (Nikon) on a PC computer. For short-term phase-contrast live-cell imaging (Figure 1A), cells were imaged in a modified Rose chamber and maintained at ∼36° C by means of an air stream incubator (Nevtek). For initial analysis of MN formation (Figure 1A), multiple monopolar cells were identified, the XYZ coordinates were recorded, and then images of individual cells were acquired every minute throughout mitosis, up to 90 minutes, using a 60x/1.4 NA Plan-Apochromatic phase contrast objective. For long-term phase-contrast live cell imaging (Figures 1D, 3A–3B, and 7A–7B), cells were imaged in 35 mm Petri dishes in a stage top incubator (Tokai Hit), with temperature set at 36° C. Ten or more MNed cells were selected and the XYZ coordinates recorded. The same number of non-MNed cells were selected by selecting one non-MNed cell within the same field of view and positioned next to the selected MNed cells. Image acquisition was performed as described above, except that images were acquired every 2–3 minutes for 17–80 hours using a 40x/0.6 NA Plan Fluor ELWD phase contrast objective. The data collected in these experiments were used to determine the ability of MNed cells to re-enter mitosis after MN formation compared to non-MNed cells (Figure 1D); the cells going through mitosis during the period of imaging were then further analyzed to determine chromosome segregation defects (Figure 3); finally, the cells imaged beyond mitosis were analyzed to measure the rates of cleavage furrow regression (Figure 7A–7C).

### Chromosome photoactivation and imaging

H2B-PAGFP PtK1cells were either grown into glass-bottom dishes (MatTek) or grown on coverslips and then transferred into a modified Rose chamber with top coverslip filled with Phenol red-free L-15 media (Gibco). The day following MN induction, cells were placed on a Nikon Eclipse Ti microscope equipped with transmitted light shutter, Lumen 200PRO fluorescence illumination system (Prior Scientific), HQ2 CCD camera (Photometrics), and ProScan automated stage (Prior Scientific). Temperature was controlled by either (for Rose chambers) an air stream incubator (Nevtek) or (for glass-bottom dishes) by a stage top incubator (Tokai Hit). Up to 40 MNed cells were initially identified, the XYZ coordinates recorded, and cells followed by phase-contrast microscopy acquiring images every 2–5 minutes with 60×/1.4 NA Plan-Apochromatic phase contrast objective lens. The cells were monitored until any of the cells reached late prophase, as determined by the level of condensation of chromosomes in the primary nucleus. At that time, imaging was interrupted to perform photoactivation of the MN, a condensed chromosome in the main nucleus (used as control), or both. Photoactivation was performed using a Mosaic Photoactivation System (Photonic Instruments/Andor) consisting of digital diaphragm optical head with micromirror array, using a 100 W Olympus (U-RFL-T) mercury lamp and a dichroic mirror that transmits light at 365–435 nm and reflects above 435 nm. One phase-contrast image of the cell of interest was acquired and, using the phase-contrast image on the screen, a region of interest was selected around the MN, around a chromosome within the main nucleus, or both, and photoactivation was achieved by illumination of the region(s) of interest through five focal planes at 0.6 μm intervals, with pulses of 100 ms at each focal plane. Imaging was resumed after photoactivation by acquiring fluorescence and phase-contrast images. Initially, fluorescence images were acquired sporadically, and mitotic events were monitored by phase-contrast imaging, until the cell reached late metaphase, at which time fluorescence and phase-contrast images were acquired simultaneously to capture chromosome segregation events involving the activated chromosomes.

## SUPPLEMENTARY MATERIALS VIDEOS



















## Abbreviations

CIN: Chromosomal Instability
LC(s): Lagging Chromosome(s)
MN/MNi: Miconucleus/Micronuclei
mnChr: micronucleus-derived chromosome
MNed: Micronucleated
pH3: phosphorylated histone H3
STLC: S-Trityl-L-cysteine
