# Supplementary material for: Chromosomes missegregated into micronuclei contribute to chromosomal instability by missegregating at the next division
Source: Oncotarget. 2019 Apr 12;10(28):2660–74. doi: 10.18632/oncotarget.26853 (PMC6505630; doi:10.18632/oncotarget.26853)
Supplement: Supplementary file 1 [file oncotarget-10-2660-s001.pdf]

Chromosomes missegregated into micronuclei contribute to chromosomal instability by missegregating at the next division

SUPPLEMENTARY MATERIALS

Supplementary Video 1: Formation of a whole chromosome-containing MN in a PtK1 cell. Time-lapse phase contrast microscopy of a PtK1 cell undergoing mitosis after STLC washout. A lagging chromosome becomes evident after anaphase onset and forms a MN upon mitotic exit. Images were acquired every minute for 90 min using a 60×/1.4 NA Plan-Apochromatic phase contrast objective. The time stamp indicates elapsed time, with 0 corresponding to the first frame. Scale bar, 5 μm. Video related to Figure 1A. See Supplemenatary_Video_1

Supplementary Video 2: MNed and non-MNed PtK1 cells re-entering mitosis. A MNed (white arrow) and a non-MNed (black arrow) cell in the same field of view were imaged by long-term phase contrast time-lapse microscopy. Images were acquired every 2 minutes for 17 hours using a 40×/0.6 NA Plan Fluor ELWD objective. The video shows a 320 minute (5.3 hours) period, during which both the MNed cell and the non-MNed cell undergo mitosis. The time stamp indicates elapsed time, with the first frame corresponding to 158 minutes. Scale bar, 5 μm. Video related to Figure 1D. See Supplementary_Video_2

Supplementary Videos 3: Example of chromosome missegregation in a MNed cell. MNed cell imaged by time-lapse phase contrast microscopy and displaying a chromosome that never aligns at the metaphase plate. After anaphase onset, the sister chromatids appear to separate from each other, but fail to move to the spindle poles. Instead, they lag near the cell equator and end up in the same MN in one daughter cell. Images were acquired every 3 minutes using a 40x/0.6 NA Plan Fluor ELWD objective. The time stamp indicates elapsed time relative to anaphase onset. Scale bar, 5 μm. Video related to Figure 3A. See Supplementary_Video_3

Supplementary Videos 4: Example of chromosome missegregation in a MNed cell. MNed cell imaged by time-lapse phase contrast microscopy and displaying undercondensation of the MN at the time when the nuclear envelope of the main nucleus breaks down. The mnChr becomes mingled with the rest of the chromosomes, but at the end of mitosis a LC becomes visible and gives rise to a new MN in one of the daughter cells upon mitotic exit. Images were acquired every 3 minutes using a 40×/0.6 NA Plan Fluor ELWD objective. The time stamp indicates elapsed time relative to anaphase onset. Scale bar, 5 μm. Video related to Figure 3B See Supplementary_Video_4.

Supplementary Video 5: Example of mitosis in a non-MNed cell with photoactivation of the entire nucleus. No chromosome segregation errors are displayed by this cell, indicating that photoactivation of histone H2B does not interfere with chromosome segregation. Near-simultaneous phase contrast (left) and GFP fluorescence (middle) images were acquired every 5 minutes using a 60x/1.4 NA Plan-Apochromatic phase-contrast objective. The video on the right was obtained by overlaying the phase contrast (red) and GFP fluorescence (green) time-lapse series. The time stamp indicates elapsed time, with 0 corresponding to the first frame. Scale bar, 5 μm. See Supplementary_Video_5

Supplementary Video 6: Mitosis in a PtK1 cell with photoactivation of both the mnChr and a nuclear chromosome. Chromatin in the MN and a chromosome in the main nucleus of a late prophase MNed H2B-PAGFP PtK1 cell were photoactivated. The video shows that the chromosome from the main nucleus segregates correctly, whereas the sister chromatids from the mnChr lag behind, ending up in the same daughter cell and in the same MN. Near-simultaneous phase contrast (left) and GFP fluorescence (middle) images were acquired every 5 minutes using a 60x/1.4 NA Plan-Apochromatic phase-contrast objective. The video on the right was obtained by overlaying the phase contrast (red) and GFP fluorescence (green) time-lapse series. The time stamp indicates elapsed time, with 0 corresponding to the first frame. Scale bar, 5 μm. Video related to Figure 4A. See Supplementary_Video_6

Supplementary Video 7: Mitosis in a PtK1 cell with photoactivation of a mnChr. Chromatin in the MN of a late prophase MNed H2B-PAGFP PtK1 cell was photoactivated. The video shows that he mnChr aligns at the metaphase plate and its sister chromatids separate at anaphase onset. However, they lag behind, co-segregate to the same daughter cell, and form two MNi upon mitotic exit. Near-simultaneous phase contrast (left) and GFP fluorescence (middle) images were acquired every 3 minutes using a 60x/1.4 NA Plan-Apochromatic phase-contrast objective. The video on the right was obtained by overlaying the phase contrast (red) and GFP fluorescence

(green) time-lapse series. The time stamp indicates elapsed time, with 0 corresponding to the first frame. Scale bar, 5 µm. Video related to Figure 4B. See Supplementary_Video_7

Supplementary Video 8: Furrow regression and tetraploidization in a MNed cell. MNed cell imaged by time-lapse phase contrast microscopy and displaying an undercondensed chromosome that becomes trapped by the cleavage furrow causing furrow regression, and hence cytokinesis failure and tetraploidy. Images were acquired every 2 minutes using a 40x/0.6 NA Plan Fluor ELWD objective. The time stamp indicates elapsed time relative to anaphase onset. Scale bar, 10 µm. Video related to Figure 7A–7B. See Supplementary_Video_8
